# Family aggregation and risk factors in substance use disorders over three generations in a nation-wide study

**DOI:** 10.1371/journal.pone.0177700

**Published:** 2017-05-17

**Authors:** Hans-Christoph Steinhausen, Helle Jakobsen, Povl Munk-Jørgensen

**Affiliations:** 1 Child and Adolescent Mental Health Centre, Capital Region Psychiatry, Copenhagen, Denmark; 2 Clinical Psychology and Epidemiology, Institute of Psychology, University of Basel, Basel, Switzerland; 3 Department of Child and Adolescent Psychiatry, University of Zurich, Neumünsterallee 9, Zurich, Switzerland; 4 Research Unit for Child and Adolescent Psychiatry, Psychiatric Hospital, Aalborg University Hospital, Aalborg, Denmark; 5 Department of Psychiatry, Odense, Denmark; Chiba Daigaku, JAPAN

## Abstract

**Objective:**

This nation-wide register-based study investigated how often substance use disorders (SUD) and co-morbid disorders occurred in affected families compared to control families.

**Method:**

A total of N = 2504 child and adolescent psychiatric participants who were born between 1969 and 1986 and were registered in the Danish Psychiatric Central Research Register (DPCRR) had a mental disorder before the age of 18 and developed SUD at some point during their life-time. In addition, N = 7472 controls without any psychiatric diagnosis before age 18 and matched for age, sex, and residential region were included. Psychiatric diagnoses of the first-degree relatives were also obtained. A family load component was assessed.

**Results:**

SUD occurred significantly more often in case families than in control families. SUD risk factors included SUD, depression, anxiety disorders, personality disorders, or conduct disorders in the family. Furthermore, male sex, more recent year of birth, and living in the capital city of Copenhagen were also significantly associated with having SUD. The family load explained 30% of the SUD manifestation in the case-probands. The findings in the total SUD group were mostly replicated in the two major subgroups of pure alcohol or multiple substance use disorders.

**Discussion:**

These findings based on a very large and representative dataset provide additional evidence for the strong family aggregation and further risk factors in SUD. The pattern of risk factors is largely the same for the total group of SUD and the major subgroups of pure alcohol and multiple substance use disorders.

## Introduction

The risk of substance use disorders (SUD) in relatives of individuals with SUD has been examined in various family-based studies. It is well established that, compared to control-probands, first-degree relatives of case-probands have elevated rates of alcohol abuse or dependence [1, 2], or an increased risk of drug disorders across the range of various substances [3–7]. In addition, various studies point to specificity of familial aggregation of the predominant drug of abuse [3, 7] suggesting that there may be risk factors both specific to particular substances and to SUD in general.

In addition, several investigations have shown that there is a high chance of having comorbid mental disorders in the offspring of parents with SUD including anxiety disorders [1, 8], depressive disorders [1], externalizing disorders [9], or any mental disorder [10]. Irrespective of family studies, the comorbidity of alcoholism with anxiety and depressive disorders has been also documented with strong cross-site consistency in four geographic communities [11]. Another epidemiological study showed that in late adolescence and young adulthood heavy and problematic use of alcohol co-occurs with both internalizing and externalizing problems [12]. Also in the relatives of probands selected for alcoholism, there is an association of alcoholism with anxiety disorders [1, 13], and it has been shown that first-degree relatives of ADHD probands were at elevated risk for SUD compared with relatives of control subjects [14].

Taking the opposite perspective, there is also evidence that various other clinical disorders frequently co-occur with SUD. This has been shown for the eating disorders with a subgroup of patients with bulimic features displaying comorbid substance abuse [15, 16]. According to a recent study in adults, the association between various childhood externalizing disorders and later substance use is strongest for childhood conduct disorders [17].

In terms of additional factors of influence, there is some evidence that sex both in the parents and the offspring may play a role in the family transmission process. However, studies diverge in findings by pointing to particular risks for either males or females, or no differences between men and women (see [10]. Data from the WHO world mental health surveys [18] indicate that males were more likely than females to have used drugs. In this large data-set, a sex–cohort interaction was observed, whereby not only younger cohorts were more likely to use all drugs, but the male–female gap was closing in more recent cohorts. In terms of age, major surveys and reviews indicate that adolescence is the key period of development of SUD [18, 19].

Furthermore, there has been a recent interest in potential risks for the development of mental disorders in children stemming from parental age at birth in terms of both biological and educational risks. Whereas advanced maternal age has been considered a reproductive risk as, for instance, in the well-documented risk of increasing frequencies of Down syndrome children, older paternal age has been hypothetically linked to an increased de novo mutations contributing to the manifestation of schizophrenia [20, 21].

In addition, various studies point to the potential role that urbanization might have for the development of mental disorders in the population. According to a recent review by Peen et al. (2010) based on 20 population surveys, significant pooled urban–rural OR were found for the total prevalence of psychiatric disorders, and for mood disorders and anxiety disorders. No significant association with urbanization was found for substance use disorders. Finally, both socioeconomic status and neighborhood deprivation have been identified as related risk factors of SUD in a large Swedish registry study [4].

This is another report from a series of matched, case-control, population-based analyses of three-generation family aggregation and associated risk factors of various mental disorders [22–27]. In the present study, we apply this approach to the analysis of substance use disorders (SUD). Specifically, we explore: (1) the family aggregation of SUD families with an affected proband compared to families of controls without childhood or adolescence diagnoses, (2) the effects of other mental disorders in the family members on case status with or without comorbid disorders, and (3) the association of age at first time diagnosis of depression with the family load. To avoid confounding effects, (4) the analyses were controlled for the impact of sex, year of birth, and degree of urbanization.

## Materials and methods

The following descriptions in this section match the general outline of previous publications based on the same study design that have focussed on disorders other than SUD [22–27]. The text reproduces in part the information already provided in these previous publications.

### Description of the dataset

The dataset of the present study contained N = 2504 case-probands with SUD, identified through the Danish Psychiatric Central Research Registry (DPCRR) [28, 29]. The DPCRR contains data on all individuals entering the public mental health system. From 1969 to 1994 only inpatient admissions were registered whereas both in- and outpatient admissions have been recorded since 1995. Only those born between 1^st^ of April 1969 and 31^st^ of December 1986 were included in the sample, which means the entire period of childhood and adolescence (0–18 years) was covered by the end of the study period December 10, 2009 when the cohort had a maximum age of 40 years. They had received an ICD-10 diagnosis [30] before age 18 and had received SUD as a main diagnosis before the age of 40.

In Denmark, each individual is given an individual number at birth in the Danish Central Civil Registration Register (DCR), thereby making it possible to cross-identify each person in various other country-wide registers. In this way, for each case-proband, three control-probands were identified in the DCR, yielding a total of 7472 using risk-set sampling; that is, each were alive and without registrations in the DPCRR at the time of case-proband ascertainment during childhood and adolescence, and were matched to case-probands on age (same year and month of birth), sex, and region of residence. Control-probands were excluded if they received any psychiatric diagnosis before age 18 but included if they received any diagnosis after age 18. Due to matching restrictions, not all case-probands had three control-counterparts for the analyses to be reported. Collectively, the case-probands and control-participants are referred to as probands.

Family members were identified through the DCR and DPCCR as previously described [31]. Lifetime data were obtained from the first registration of any mental disorder and the maximum period of observation for the diagnostic ascertainment of relatives via the DPCRR was 40 years. Registry diagnoses of SUD were defined according to ICD-8 [32] criteria (code 303 and 304) until 1994, then, as of 1995, according to ICD-10 [30] criteria (code F1x). To study the role of other mental disorders in the family aggregation, the following additional diagnoses were also considered in the analyses: Bipolar disorders (BP, ICD-8 code 296; ICD-10 code F30-31), depression (DEP, ICD-8 code 300.49; ICD-10 code F32-33 and 34.1), anxiety disorders (ANX, ICD-8 code 300.0, 300.2; ICD-10 code F40, F41, F93.0–93.2), eating disorders (ED, ICD-8 code 306.5; ICD-10 code F50), personality disorders (PERS, ICD-8 code 301; ICD-10 code F60), and conduct disorder (ICD-8 code 308.1–308.2; ICD-10 code F90.1, 91, 92). Furthermore, the subgroups of SUD were defined as alcoholism (ALC, ICD-8 code 303; ICD-10 code F10), abuse of opioids (ICD-8 code 304.09, 304.19; ICD-10 code F11), abuse of cannabinoids (ICD-8 code 304.59; ICD-10 code F12), abuse of sedatives or hypnotics (ICD-8 code 304.29, 304.39; ICD-10 code F13), abuse of cocaine (ICD-8 code 304.49; ICD-10 code F14), abuse of stimulants (ICD-8 code 304.69; ICD-10 code F15) and abuse of hallucinogens (ICD-8 code 304.79; ICD-10 code F16). Multiple SUD (MULT) was defined as ICD-10 code F19 or when a person had two or more of the defined SUD diagnoses.

### Statistical analyses

Chi-square tests were used to determine whether SUD occurred more often in the relatives of case-probands compared to relatives of controls, and whether SUD was present more often in families (i.e., when the family was treated as a single unit of analysis) of cases than in families of controls. Effect sizes were assessed by Cramer`s *V* for comparisons of frequencies of comorbid disorders. Coefficients ranging from 0 to 0.1 were considered very small, from 0.1 to 0.3 small, from 0.3 to 0.5 medium, and ≥0.5 large.

Conditional logistic regression was applied to determine if the illness status of family members increased the risk of the disease in the case-probands more strongly than in the control-probands. The indicator variables examined were SUD and other mental disorders in family members. If data from a family member were missing the value of the variable was 0, indicating the family member was assumed to be unaffected. Since this method takes matching into account, the matched variables were not included in the analysis. All variables were included as categorical variables.

Multinomial logistic regression analysis was used for the comparison of risk factors originating from the various mental disorders in family members in three different groups: Case-probands with SUD and any other mental disorders, case-probands with pure SUD (i.e. without any comorbidity), and the group of control-probands without SUD. The group of control-probands was used as the reference group, i.e. the presence of family disorders in both the comorbid SUD group and the pure SUD group were compared to the controls. The risk was measured as relative risk ratios (RRR).

Mixed logistic regression was used to estimate a family load measured as a random effect showing the dependence among family members in relation to how often each family developed SUD. A model including a random effect is used when a dataset is divided into groups–in this case into families. In the model, each family has its own intercept and the random effect is the estimated standard deviation (SD) in the intercept on the logarithmic scale. For instance, consider a mixed logistic regression model for a matched case-control study with families of three generations which has a random effect of SD = 0.5. This means that members of a family which is one standard deviation above the mean have the odds of getting SUD which are 65% [since exp(0.5) = 1.65] higher than members of an average family.

The random effect was examined by group (namely, cases and controls) and was divided for different age at diagnosis, namely up to 18 years, and age 18 or above at diagnosis of SUD in the case-probands. Furthermore, the regression analysis included the matched explanatory variables, i.e., sex, year of birth, month of birth, and region of residence at the index time of the case-probands. In the analyses, the variable region of residence was defined as the hospital, where the case-proband of the family received the first diagnosis. This value was copied to all the case family-members and to the matched control families assuming that the case-proband attended the nearest hospital and that the family members lived at the same place as the proband, meaning the choice of hospital would reflect the region of residence. The variable “region of residence” was converted into a dichotomous variable comparing the capital city of Copenhagen to all other regions. Sex, month of birth, and region of residence were included as categorical variables, while year of birth was included as a continuous variable.

Cox regression with shared frailty was applied to investigate whether case family members had a greater risk over time of developing SUD than control family members, i.e. the probands were excluded from the analysis. A Cox model is a survival model and it is a function of the hazard rate, which is the risk over time of experiencing a certain event such as SUD. A Cox model with shared frailty includes a random effect named a frailty, which describes the effect of unknown elements not included as parameters in the model. The family load component is estimated as a random effect (frailty), i.e., it describes the dependence or lack of such between the families of the study. The frailty does not vary within families, but rather between these. The frailty is assumed to follow the gamma distribution with a mean value of 1 and variance θ. The purpose of this approach is to estimate the effect of the explanatory variables while also estimating θ.

Case status was included as a variable in the model in order to compare the risk of SUD over time in case family members vs. control family members. The analysis also includes the explanatory variables sex, year and month of birth, and region of residence, that were used in the matching of the probands. The latter was converted into a dichotomous variable comparing the capital of Copenhagen to all other regions. Sex, month of birth and region of residence were included as categorical variables, whereas year of birth was included as a continuous variable. All analyses were carried out by using the statistical software programs Stata version 13.1 [33] and R version 3.0.3 [34].

## Results

Sample sizes, sex distributions, and the observation periods in the stratified case- and control samples are shown in Table 1. In both case and control populations the maximum average observational period was 40.69 years. For the case-probands, the mean observation time amounted to 20.73 years. In the total group of N = 2504 case-probands there were N = 525 case-probands with a pure alcohol use disorder and N = 1285 case-probands with multiple substance use disorders. In addition, there were N = 478 case-probands with pure cannabinoid use disorder, and N = 39 case-probands with pure opioid use disorders. The remaining case-probands were dispersed across the categories of abuse of pure sedatives or hypnotics (N = 24), pure cocaine (N = 21), pure stimulants (N = 81), pure hallucinogens (N = 17), pure tobacco (N = 4), pure volatile solvents (N = 7), and unspecified drug dependency (N = 23). Only the alcohol use disorders (ALC) and the multiple substance use disorders (MULT) were considered for further sub-analyses because there were enough case-probands with affected family members in these groups to investigate the family aggregation.

**Table 1:** Demographic characteristics of the subjects.

	N (%)			Observation time in years*		
	Total	Males	Females	Mean	SD	Range
Case families						
Probands	2504	1513 (60.42)	991 (39.58)	20.73	4.70	12.37–39.76
Fathers	2406	2406 (100)	0 (0)	35.92	9.75	0.10–40.69
Mothers	2492	0 (0)	2492 (100)	37.67	7.89	0.27–40.69
Siblings	2488	1272 (51.13)	1216 (48.87)	28.50	8.16	0.00–40.69
Offspring	697	374 (53.66)	323 (46.34)	10.51	3.88	0.02–22.87
Total	10587	5565 (52.56)	5022 (47.44)	29.32	11.20	0.00–40.69
Control families						
Probands	7472	4515 (60.43)	2957 (39.57)	29.34	4.98	12.14–40.69
Fathers	7330	7330 (100)	0 (0)	39.03	5.63	0.73–40.69
Mothers	7443	0 (0)	7443 (100)	39.91	3.79	1.11–40.69
Siblings	9991	5153 (51.58)	4838 (48.42)	28.71	8.00	0.00–40.69
Offspring	2095	1075 (51.31)	1020 (48.69)	9.43	3.50	0.00–23.06
Total	34331	18073 (52.64)	16258 (47.36)	32.31	9.72	0.00–40.69

*time from birth or 1^st^ of April 1969 until date of first SUD diagnosis, date of death or end of study the 10^th^ of December 2010

The frequencies of SUD in families of case and control-probands by class of relative in the total group and in the two major subgroups of SUD are shown in Table 2. SUD was only counted among case-parents and siblings if the diagnosis was given before the first SUD diagnosis in the case-proband of the family. For the control parents and siblings, the diagnosis was given before the date of SUD of the matched case-proband. The SUD diagnosis for the offspring was also counted if it appeared after the SUD diagnosis of the case-proband. The odds of case-proband family relatives having SUD in the total group were 5.96 times those for relatives of control-probands, thus supporting the assumption of familial aggregation of SUD. The differences were significant for parents and siblings. More specifically, the odds of SUD were 4.51 times higher in fathers of case-probands compared to fathers of control-probands, 7.69 times higher in mothers of case-probands compared to mothers of control-probands and 7.72 times higher in siblings of case-probands compared to siblings of control-probands. There were too few offspring with SUD to say anything definite about the amount of SUD in cases compared to controls. All the effect sizes were very small to small on each level of the family comparisons.

**Table 2:** The distribution of SUD and age at diagnosis in case and control families.

	TOTAL N(%)			ALC N(%)			MULT N(%)		
	SUD not present		SUD present	SUD not present		SUD present	SUD not present		SUD present
Case families									
Probands	0 (0.00)		2504 (100.00)	0 (0.00)		525 (100.00)	0 (0.00)		1285 (100.00)
Fathers	2114 (87.86)		292 (12.14)	470 (91.62)		43 (8.38)	1164 (95.02)		61 (4.98)
Mothers	2258 (90.61)		234 (9.39)	480 (91.78)		43 (8.22)	1218 (95.31)		60 (4.69)
Siblings	2415 (97.07)		73 (2.93)	541 (97.30)		15 (2.70)	1167 (93.96)		75 (6.04)
Offspring	694 (99.57)		3 (0.43)	212 (100.00)		0 (0.00)	307 (99.68)		1 (0.32)
Total	7481 (70.66)		3106 (29.34)	1703 (73.12)		626 (26.88)	3856 (72.24)		1482 (27.76)
Control families									
Probands	7372 (98.66)		100 (1.34)	1564 (99.62)		6 (0.38)	3817 (99.45)		21 (0.55)
Fathers	7112 (97.03)		218 (2.97)	1490 (96.75)		50 (3.25)	3736 (99.28)		27 (0.72)
Mothers	7344 (98.67)		99 (1.33)	1543 (98.66)		21 (1.34)	3794 (99.32)		26 (0.68)
Siblings	9952 (99.61)		39 (0.39)	2101 (99.72)		6 (0.28)	5161 (99.59)		21 (0.41)
Offspring	2093 (99.90)		2 (0.10)	632 (100.00)		0 (0.00)	1014 (99.80)		2 (0.20)
Total	33873 (98.67)		458 (1.33)	7330 (98.88)		83 (1.12)	17522 (99.45)		97 (0.55)
	OR	95% CI	V^1^	OR	95% CI	V^1^	OR	95% CI	V^1^
Cases vs. controls									
Fathers	4.51**	3.75–5.42	0.18	2.73**	1.79–4.16	0.11	7.25**	4.57–11.51	0.14
Mothers	7.69**	6.02–9.82	0.19	6.58**	3.84–11.30	0.17	7.19**	4.50–11.49	0.14
Siblings	7.72**	5.21–11.44	0.11	9.72**	3.73–25.32	0.11	15.78**	9.61–25.93	0.18
Offspring	4.52	0.75–27.15	0.03	-	-	-	1.65	0.15–18.28	0.01
Total	5.96**	5.20–6.82	0.16	4.44**	3.28–6.02	0.12	9.21**	7.03–12.06	0.15

^1^ Cramer's V

* = p<0.05

** = p<0.001

These findings were mirrored in the two subgroups ALC and MULT. For ALC, the odds of case-proband family relatives having ALC before the date of ALC of the case-proband were 4.44 times those for relatives of control-probands, whereas for MULT, the odds of case-proband family members having MULT before the date of MULT of the case-proband were 9.21 times those of control-probands. Thus, the family aggregation of SUD in total was also reflected in the two major subgroups ALC and MULT. As Table 2 shows, the odds of ALC and MULT were significantly higher among fathers, mothers, and siblings of case-probands compared to family members of control-probands. As in the total SUD sample, the effect sizes again were very small to small on each level of the family comparisons in the ALC and MULT subgroups.

As Table 3 shows, the vast majority of case-probands had comorbid disorders, most common was PERS followed by DEP, CD, and ANX and less frequently by ED and BP. Information on the frequencies of the six co-morbid disorders by class of relative across the case and control-families and the subgroups is provided in Table 4. Again, diagnoses among case-parents and siblings were only counted if a diagnosis was given before the SUD diagnosis in the case-proband of the family, and for the control-parents and siblings the diagnosis had to be given before the date of SUD of the matched case-proband. The diagnoses for the offspring were counted also if they appeared after the SUD diagnosis of the case-proband.

**Table 3:** Comorbid diagnoses among case and control-probands.

	N(%)	
	Cases	Controls
Probands with pure SUD	250 (9.98)	26 (0.35)
Probands with comorbid SUD	2254 (90.02)	74 (0.99)
Comorbid disorders among probands with SUD		
BP	101 (4.48)	4 (5.41)
DEP	539 (23.91)	28 (37.84)
ANX	293 (13.00)	10 (13.51)
ED	171 (7.59)	1 (1.35)
PERS	1195 (53.02)	14 (18.92)
CD	350 (15.53)	0 (0.00)

**Table 4:** The distribution of comorbid disorders in case and control families.

	TOTAL N (%)			ALC N (%)			MULT N (%)		
	Case families	Control families	V^1^	Case families	Control families	V^1^	Case families	Control families	V^1^
BP									
Fathers	27 (1.12)	47 (0.64)	0.02*	6 (1.17)	10 (0.65)	0.03	15 (1.22)	30 (0.80)	0.02
Mothers	49 (1.97)	57 (0.77)	0.05**	11 (2.10)	16 (1.02)	0.04	28 (2.19)	25 (0.65)	0.07**
Siblings	5 (0.20)	9 (0.09)	0.01	4 (0.72)	3 (0.14)	0.05*	1 (0.08)	5 (0.10)	0.00
Offspring	0 (0.00)	0 (0.00)	-	0 (0.00)	0 (0.00)	-	0 (0.00)	0 (0.00)	-
Total	81 (1.00)	113 (0.42)	0.03**	21 (1.16)	29 (0.50)	0.04*	44 (1.09)	60 (0.44)	0.04**
DEP									
Fathers	52 (2.16)	77 (1.05)	0.04**	9 (1.75)	16 (1.04)	0.03	28 (2.29)	47 (1.25)	0.04*
Mothers	120 (4.82)	113 (1.52)	0.09**	24 (4.59)	27 (1.73)	0.08*	52 (4.07)	58 (1.52)	0.08**
Siblings	54 (2.17)	54 (0.54)	0.07**	12 (2.16)	20 (0.95)	0.05*	30 (2.42)	22 (0.42)	0.09**
Offspring	1 (0.14)	5 (0.24)	0.01	0 (0.00)	2 (0.32)	0.03	0 (0.00)	2 (0.20)	0.02
Total	227 (2.81)	249 (0.93)	0.07**	45 (2.49)	65 (1.11)	0.05**	110 (2.71)	129 (0.94)	0.06**
ANX									
Fathers	26 (1.08)	28 (0.38)	0.04**	4 (0.78)	6 (0.39)	0.02	14 (1.14)	14 (0.37)	0.04*
Mothers	98 (3.93)	82 (1.10)	0.09**	23 (4.40)	21 (1.34)	0.09**	50 (3.91)	38 (0.99)	0.10**
Siblings	21 (0.84)	26 (0.26)	0.04**	4 (0.72)	5 (0.24)	0.03	12 (0.97)	15 (0.29)	0.04*
Offspring	2 (0.29)	3 (0.14)	0.01	0 (0.00)	1 (0.16)	0.02	1 (0.32)	1 (0.10)	0.02
Total	147 (1.82)	139 (0.52)	0.06**	31 (1.72)	33 (0.56)	0.05**	77 (1.90)	68 (0.49)	0.07**
ED									
Fathers	0 (0.00)	0 (0.00)	-	0 (0.00)	0 (0.00)	-	0 (0.00)	0 (0.00)	-
Mothers	9 (0.36)	8 (0.11)	0.03*	3 (0.57)	0 (0.00)	0.07*	6 (0.47)	6 (0.16)	0.03
Siblings	19 (0.76)	30 (0.30)	0.03*	2 (0.36)	7 (0.33)	0.00	12 (0.97)	13 (0.25)	0.05*
Offspring	2 (0.29)	3 (0.14)	0.01	2 (0.94)	1 (0.16)	0.06	0 (0.00)	2 (0.20)	0.02
Total	30 (0.37)	41 (0.15)	0.02**	7 (0.39)	8 (0.14)	0.02	18 (0.44)	21 (0.15)	0.03*
PERS									
Fathers	191 (7.94)	147 (2.01)	0.14**	32 (6.24)	31 (2.01)	0.11**	115 (9.39)	76 (2.02)	0.17**
Mothers	288 (11.56)	153 (2.06)	0.20**	60 (11.47)	39 (2.49)	0.18**	155 (12.13)	80 (2.09)	0.21**
Siblings	90 (3.62)	70 (0.70)	0.10**	24 (4.32)	19 (0.90)	0.11**	50 (4.03)	34 (0.66)	0.12**
Offspring	3 (0.43)	4 (0.19)	0.02	3 (1.42)	1 (0.16)	0.08	0 (0.00)	2 (0.20)	0.02
Total	572 (7.08)	374 (1.39)	0.15**	119 (6.60)	90 (1.54)	0.13**	320 (7.90)	192 (1.39)	0.16**
CD									
Fathers	0 (0.00)	2 (0.03)	0.01	0 (0.00)	0 (0.00)	-	0 (0.00)	0 (0.00)	-
Mothers	0 (0.00)	0 (0.00)	-	0 (0.00)	0 (0.00)	-	0 (0.00)	0 (0.00)	-
Siblings	23 (0.92)	16 (0.16)	0.05**	6 (1.08)	3 (0.14)	0.07*	16 (1.29)	10 (0.19)	0.07**
Offspring	16 (2.30)	7 (0.33)	0.09**	3 (1.42)	0 (0.00)	0.10*	9 (2.92)	6 (0.59)	0.09*
Total	39 (0.48)	25 (0.09)	0.04**	9 (0.50)	3 (0.05)	0.05**	25 (0.62)	16 (0.12)	0.04**

Fishers exact test

* = p<0.05

** = p<0.001

^1^ Cramer's V

In the total SUD sample, DEP, ANX, and PERS were significantly more common in all relatives of case-probands, except the offspring compared to the relatives of control-probands. Furthermore, BD were significantly more common in parents of case-probands compared to the parents of controls, ED were significantly more common in mothers and siblings of case-probands compared to the controls, and CD were significantly more common in siblings and offspring of case-probands compared to the controls. In terms of the effect sizes, all associations were in the very small to small range.

Subsequent analyses in the two subgroups with either ALC or MULT showed mostly similar findings. In contrast to the comparisons in the total SUD sample, Fishers exact tests between case and control family members in the ALC subgroup showed no significant differences for parents with bipolar disorders whereas more case siblings than control siblings had bipolar disorders. Also, the differences between the number of case and control fathers with depression or anxiety disorders and the differences between the number of case and control siblings with anxiety disorders or eating disorders were no longer significant in the ALC subgroup, most likely due to the smaller sample size making the detection of differences more difficult. In the ALC group there was also no significant differences between the total number of family members with eating disorders in case vs. control families. The respective comparisons in the MULT subgroup also revealed no significant differences for fathers with bipolar disorders and mothers with eating disorders.

**Fig 1:**
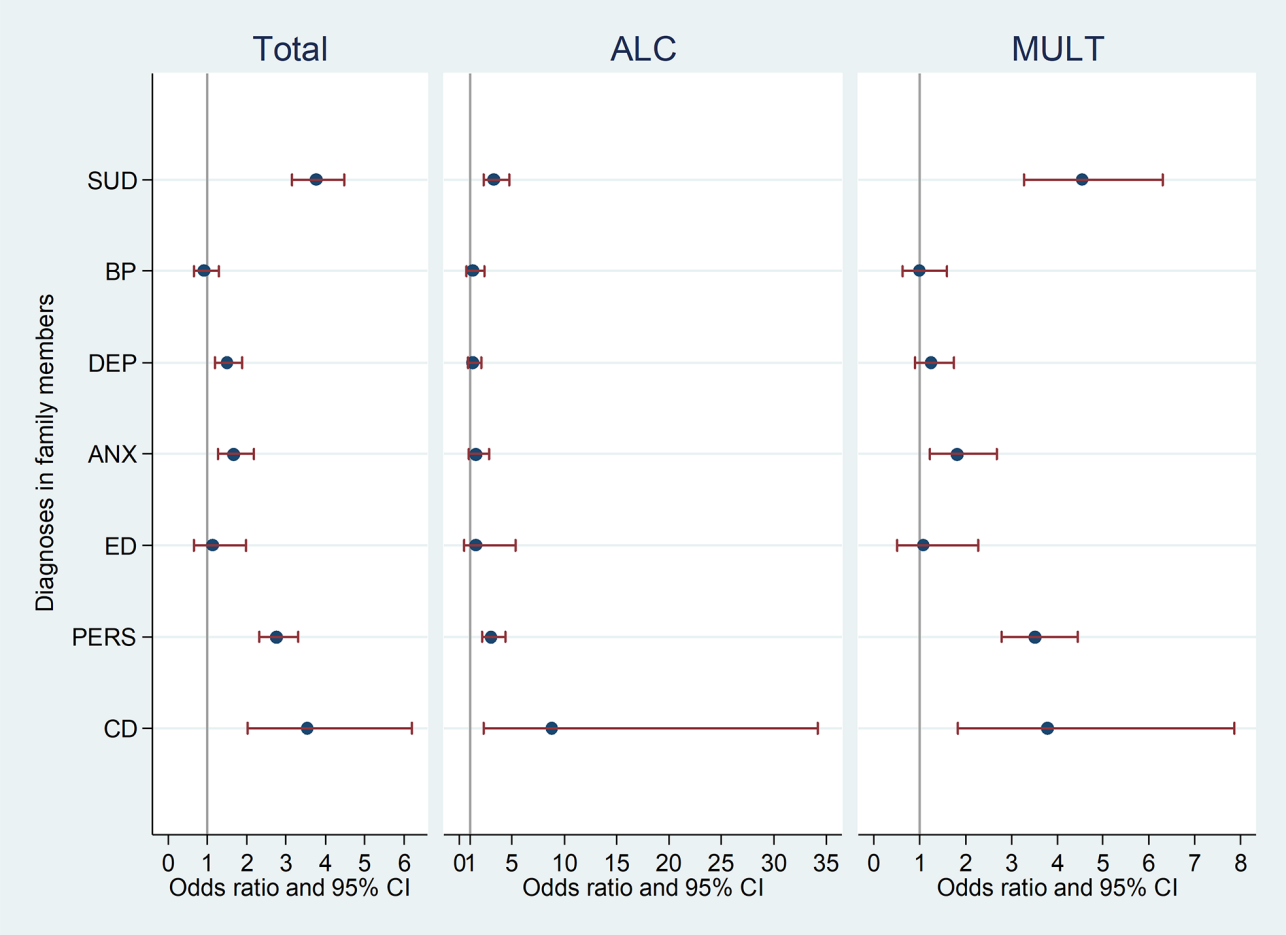
Associations of SUD in the case-probands versus the control-probands with SUD and other mental SUDs in first-degree family members in the total sample and the pure alcohol (ALC) and the multiple (MULT) substance use disorders. SUD = Substance use disorders; BD = Bipolar disorders; DEP = Depression; ANX = Anxiety disorders; ED = Eating disorders; PERS = Personality disorders; CD = Conduct disorders.

Findings from the conditional logistic regression analysis determining the association of SUD, ALC, or MULT in case-probands vs. control-probands with either SUD or other mental disorders in family members are illustrated in Fig 1. As in the previous analyses, SUD was only counted in parents and siblings if it appeared before the SUD diagnosis of the case-proband. Compared to the control- probands, in the total sample SUD in the case-probands was significantly associated with SUD (OR = 3.76, p<0.001, 95% CI = 3.16–4.49), DEP (OR = 1.51, p<0.001, 95% CI = 1.20–1.89), ANX (OR = 1.66, p<0.001, 95% CI = 1.26–2.19), PERS (OR = 2.76, p<0.001, 95% CI = 2.31–3.30), and CD (OR = 3.55, p<0.001, 95% CI = 2.04–6.20) but not with BP (OR = 0.92, p = n.s., 95% CI = 0.65–1.29) and ED (OR = 1.14, p = n.s., 95% CI = 0.66–1.99) in family members.

In the ALC subgroup, the associations with SUD (only ALC was counted; OR = 3.30, p<0.001, 95% CI = 2.28–4.76), PERS (OR = 3.08, p<0.001, 95% CI = 2.17–4.39) and CD (OR = 8.87, p<0.05, 95% CI = 2.30–34.18) were significant, whereas BP (OR = 1.23, p = n.s., 95% CI = 0.64–2.39), DEP (OR = 1.29, p = n.s., 95% CI = 0.81–2.05), ANX (OR = 1.58, p = n.s., 95% CI = 0.90–2.79) and ED (OR = 1.59, p = n.s., 95% CI = 0.48–5.31) were not. The confidence intervals for CD in the ALC group were rather wide as very few family members in this group had CD, making the result less precise. There were significant associations with SUD in the MULT subgroup (only MULT was counted; OR = 4.54, p<0.001, 95% CI = 3.28–6.30), ANX (OR = 1.82, p<0.05, 95% CI = 1.23–2.68), PERS (OR = 3.52, p<0.001, 95% CI = 2.78–4.45) and CD (OR = 3.79, p<0.001, 95% CI = 1.83–7.87), whereas BP (OR = 1.00, p = n.s., 95% CI = 0.63–1.59), DEP (OR = 1.25, p = n.s., 95% CI = 0.90–1.75) and ED (OR = 1.08, p = n.s., 95% CI = 0.51–2.28) were not significant.

Table 5 shows the results of multinomial logistic regression considering SUD as a comorbid condition (N = 2254) in the case-probands vs. a pure condition without any further comorbidity (N = 250), where the controls without SUD are the reference group. In this analysis, parental age at birth of the proband and the presence of SUD and other mental disorders in family members served as risk factors, and the model was adjusted for the matched variables. Both family SUD and the variable marking other mental disorders among family-members were measured by binary variables with a value of 1 if a disorder was present before the date of SUD of the case-proband and otherwise zero. These analyses were performed only for the total SUD group because both in the ALC and in the MULT subgroup the number of individuals with a non-comorbid pure SUD diagnosis were too small for reliable analyses.

**Table 5:** Multinomial logistic regression with three groups of probands as the outcome variable (case-probands having either comorbid or pure SUD and SUD-free controls as the reference group). The model is adjusted for the matched variables.

	RRR	SE	P	95% CI
*Controls*	*Reference group*			
*Comorbid SUD*				
Maternal age at birth				
Age<35	Reference			
Age≥35	0.89	0.10	n.s.	0.71–1.12
Paternal age at birth				
Age<35	Reference			
Age≥35	1.05	0.08	n.s.	0.91–1.21
Family SUD				
No	Reference			
Yes	4.62	0.57	<0.001	3.63–5.88
Family BP, DEP, ANX, ED, PERS or CD				
No	Reference			
Yes	3.19	0.25	<0.001	2.73–3.71
Interaction between family SUD and family BP, DEP, ANX, ED, PERS or CD	0.64	0.11	<0.05	0.46–0.90
*Pure SUD*				
Maternal age at birth				
Age<35	Reference			
Age≥35	0.67	0.22	n.s.	0.35–1.30
Paternal age at birth				
Age<35	Reference			
Age≥35	0.94	0.18	n.s.	0.64–1.37
Family SUD				
No	Reference			
Yes	4.90	1.21	<0.001	3.02–7.96
Family BP, DEP, ANX, ED, PERS or CD				
No	Reference			
Yes	1.51	0.36	n.s.	0.95–2.40
Interaction between family SUD and family BP, DEP, ANX, ED, PERS or CD	0.32	0.15	<0.05	0.13–0.78

RRR = Relative risk ratio (the exponentiated coefficients from the multinomial logistic regression model); SUD = Substance Use Disorders

The results demonstrated that if at least one family member had SUD, then the relative risk for a case-proband getting comorbid SUD was expected to increase by a factor of 4.62 compared to the controls, given the other variables in the model were held constant. The results also showed that if a family member had SUD, then the relative risk for a case-proband getting pure SUD was expected to increase by a factor of 4.90 compared to the controls, given the other variables in the model were held constant. A Wald test was performed to test whether or not the effect of family SUD in predicting comorbid SUD among case-probands vs. controls-probands equals the effect of family SUD in predicting pure SUD among case-probands vs. control-probands. The test showed that the effects were not statistically different from each other (χ^2^ = 0.06, p = 0.81).

In the same way, the effect of one or more of the diagnoses BP, DEP, ANX, ED, PERS or CD in the family was studied. A Wald test showed that the effect of one or more of the diagnoses BP, DEP, ANX, ED, PERS or CD in the family members in predicting comorbid SUD among case- probands vs. controls-probands was statistically different (χ^2^ = 9.71, p<0.05) from the effect of one or more of the defined diagnoses in the family members in predicting pure SUD among case-probands vs. control-probands.

An interaction term was included in the model in order to test the extra effect of having both SUD and one or more of the diagnoses BP, DEP, ANX, ED, PERS or CD in the family-members. In the group of case-probands with comorbid SUD, the exponential coefficient for the interaction was 0.64. This means that that if SUD and one or more of the diagnoses BP, DEP, ANX, ED, PERS or CD were present in a family, then the relative risk of the case-proband getting comorbid SUD was expected to increase by a factor of 4.62*3.19*0.64 = 9.43 compared to the controls, given the other variables in the model were held constant. In the same way, if both SUD and one or more of the other defined disorders were present in a family, the relative risk for the case-proband getting pure SUD was expected to increase by a factor of 4.90*1.51*0.32 = 2.37 compared to the controls, if the other variables in the model are held constant. A Wald test showed that the effect of the interaction in predicting comorbid SUD among case-probands vs. control-probands was not significantly different (χ^2^ = 2.34, p = 0.13) from the effect of the interaction in predicting pure SUD among case-probands vs. control-probands.

Table 6 shows the results of mixed logistic regression, which has the purpose of estimating the family load. The matched variables were included in order to take the matching into account. Male sex, and more recent year of birth were significant additional risk factors in the total group and in the ALC and MULT subgroups whereas living in the capital city of Copenhagen was a significant additional risk factor only in the total and the MULT groups. Being born in August, September, October or November also had significant p-values in the total group, but the confidence intervals were very close to, 1 and had a range almost similar to the confidence intervals for the other non-significant months of birth. Thus, the results do not necessarily indicate that being born in these months is any riskier than being born in January (the reference group). The random effect contributed with 30 percent of the total variance in the total group and a likelihood-ratio test of a contribution to variance equal to zero showed that a model with a random effect explained more of the data than a model without a random effect (χ^2^ = 443.23, p <0.001). The random effect contributed to 19% of the total variance in the ALC group, and 27% in the MULT group. A likelihood-ratio test of a contribution to variance equal to zero showed that that a model with a random effect explained more of the data than a model without a random effect in both the ALC and the MULT subgroups (ALC: χ^2^ = 32.54, p<0.001; MULT: χ^2^ = 141.79, p<0.001).

**Table 6:** Mixed logistic regression with the purpose of estimating the family load, which is measured as a random effect.

	TOTAL			ALC			MULT		
	OR	SE	95% CI	OR	SE	95% CI	OR	SE	95% CI
Sex									
Female	1.00		Reference	1.00		Reference	1.00		Reference
Male	1.57**	0.06	1.45–1.69	1.30*	0.11	1.10–1.53	1.59**	0.09	1.42–1.79
Month of birth									
January	1.00		Reference	1.00		Reference	1.00		Reference
February	0.96	0.10	0.79–1.18	0.87	0.19	0.57–1.33	0.99	0.14	0.74–1.32
March	1.00	0.10	0.83–1.22	0.95	0.20	0.63–1.44	0.87	0.13	0.66–1.15
April	1.03	0.10	0.85–1.25	1.14	0.22	0.78–1.68	0.87	0.13	0.66–1.16
May	1.13	0.11	0.93–1.36	1.16	0.23	0.78–1.71	0.98	0.14	0.74–1.29
June	1.15	0.11	0.95–1.39	1.05	0.22	0.70–1.58	0.94	0.13	0.71–1.25
July	1.09	0.11	0.90–1.31	1.16	0.23	0.78–1.71	0.97	0.14	0.74–1.28
August	1.22*	0.12	1.01–1.47	1.10	0.23	0.73–1.66	1.11	0.15	0.84–1.45
September	1.22*	0.12	1.01–1.48	1.10	0.22	0.74–1.64	1.13	0.16	0.86–1.48
October	1.25*	0.12	1.04–1.51	1.12	0.23	0.74–1.69	1.06	0.15	0.80–1.40
November	1.27*	0.12	1.05–1.54	0.91	0.20	0.58–1.40	1.33*	0.18	1.02–1.75
December	1.21	0.12	1.00–1.47	0.98	0.21	0.65–1.49	1.13	0.16	0.85–1.49
Year of birth	1.02**	0.00	1.02–1.02	1.02**	0.00	1.01–1.02	1.04**	0.00	1.04–1.04
Region of residence									
City of Copenhagen	1.00		Reference	1.00		Reference	1.00		Reference
Other regions	0.87*	0.04	0.79–0.97	0.90	0.09	0.74–1.09	0.85*	0.06	0.73–0.98
	RE	SE	95% CI	RE	SE	95% CI	RE	SE	95% CI
Standard deviation	1.18	0.04	1.10–1.26	0.87	0.09	0.70–1.07	1.12	0.06	1.00–1.25
Contribution to variance	0.30	0.01	0.27–0.32	0.19	0.03	0.13–0.26	0.27	0.02	0.23–0.32

* = p<0.05

** = p<0.001

RE = random effect results

**Fig 2:**
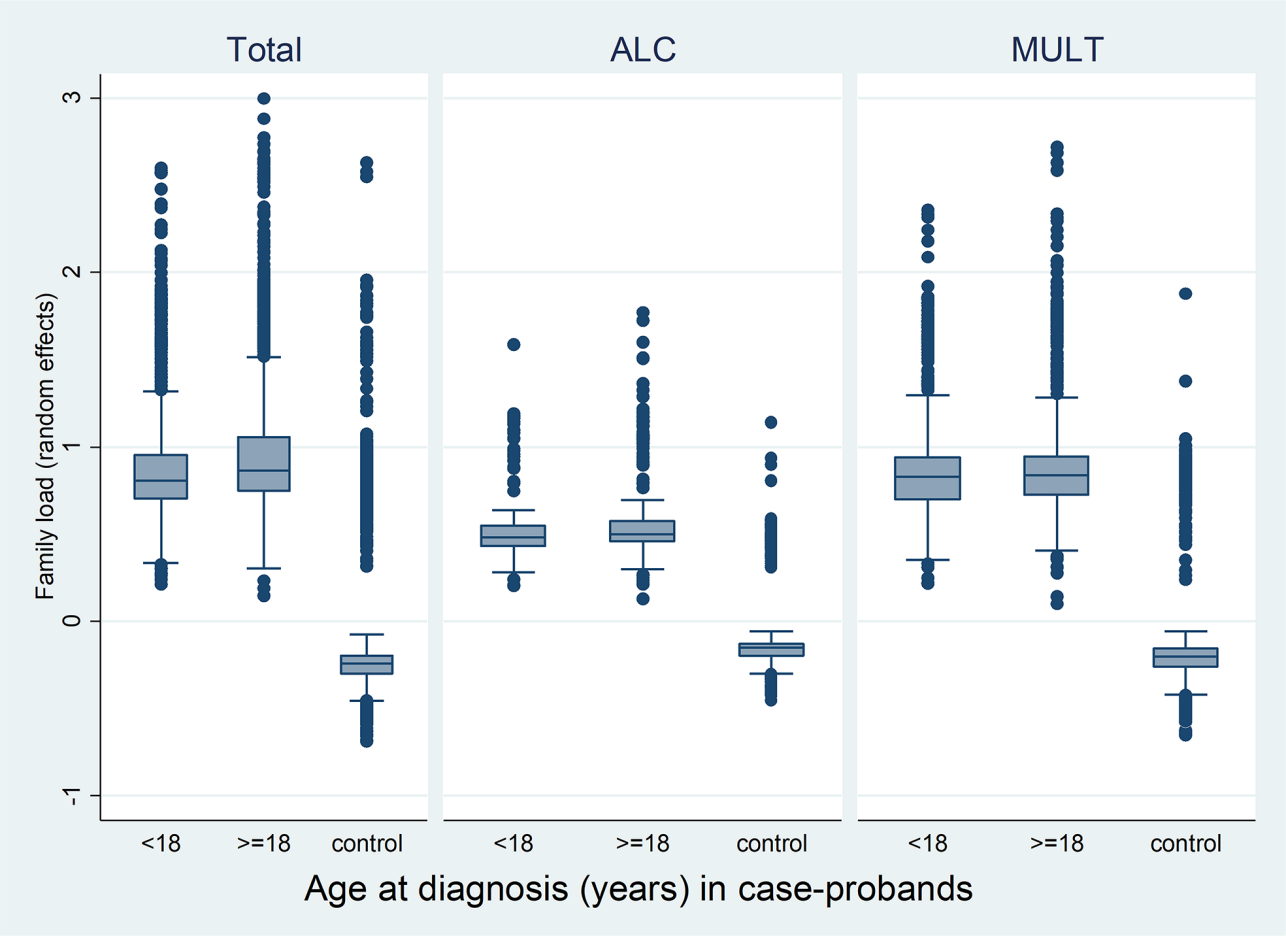
Family load components in case-probands by age at diagnosis and controls in the total sample and the pure alcohol (ALC) and the multiple (MULT) substance use disorders.

The box plot in Fig 2 displays the family load components estimated based on the mixed logistic regression model from Table 6. Year of birth, month of birth, sex and region of residence made up the fixed effects of the mixed logistic regression model, while the random effect reflected the correlation between family members, i.e. the risk level of SUD for each family. The family load was defined as the random effect which is shown in Fig 2. It can be seen that in both the total sample and in the ALC and MULT subgroups case families had a significantly higher family load component than control families meaning that family aggregation explains a larger part of the variance in case families than in control families. The two-sample Wilcoxon rank-sum test for testing the family load among males and females separate for the case and the control-probands showed that in the total sample there were significant differences in the family load between males and females among both cases and control-probands, with a higher load in females (Cases: Females N = 991, rank sum = 1301192, expected = 1241227.5 vs. males N = 1513, rank sum = 1835068, expected = 1895032.5; z = 3.39, p<0.001. Controls: Females N = 2957, rank sum = 11855517, expected = 11048831 vs. males N = 4515, rank sum = 16063611, expected = 16870298; z = 8.85, p<0.001).

The same test in the ALC subgroup showed no significant differences in the family load between male and female probands in both the case and the control group (Cases: Females N = 235, rank sum = 61214, expected = 61805 vs. males N = 290, rank sum = 76861, expected = 76270; z = -0.34, p = 0.73. Controls: Females N = 704, rank sum = 568237, expected = 552992 vs. males N = 866, rank sum = 664998, expected = 680243; z = 1.71, p = 0.09). In the MULT subgroup, the two-sample Wilcoxon rank-sum test had a result similar to the result in the total SUD sample, i.e. a significantly higher load in females (Cases: Females N = 481, rank sum = 329788.5, expected = 309283 vs. males N = 804, rank sum = 496466.5, expected = 516972; z = 3.19, p<0.05. Controls: Females N = 1436, rank sum = 2939918, expected = 2756402 vs. males N = 2402, rank sum = 4427123, expected = 4610639; z = 5.52, p<0.001).

The two-sample Wilcoxon rank-sum test was also used to test whether there was a difference between the family load of case-probands with the diagnosis of SUD before age 18 vs. case-probands with the diagnosis of SUD at age 18 or later but before the end of the study. The test demonstrated a significantly higher load in cases with diagnosis at or after age 18 (diagnosis of SUD before age 18: N = 1032, rank sum = 1176391.5, expected = 1292580 vs. diagnosis of SUD at age 18 or later: N = 1472, rank sum = 1959868.5, expected = 1843680; z = -6.53, p<0.001). The same test in the subgroup ALC also showed a significantly higher load in cases with diagnosis at or after age 18 (diagnosis of SUD before age 18: N = 188, rank sum = 44623, expected = 49444 vs. diagnosis of SUD at age 18 or later: N = 337, rank sum = 93452, expected = 88631; z = -2.89, p<0.05), whereas the test in the subgroup MULT did not show any significant differences (diagnosis of SUD before age 18: N = 526, rank sum = 333230.5, expected = 338218 vs. diagnosis of SUD at age 18 or later: N = 759, rank sum = 493024.5, expected = 488037; z = -0.762, p = 0.446).

The question of whether or not case family members had a greater risk over time of developing SUD than control family members was investigated using Cox regression with shared frailty. As described above, this method estimates the family load component as a random effect in a similar way to mixed logistic regression. However, in this analysis the family load component (frailty) is estimated in relation to the time of SUD diagnosis. All individuals were followed from birth or April 1 1969 until date of diagnosis of SUD, date of death, or December 10 2009 where, data was censored if SUD or death had not occurred. Findings are shown in Table 7. The case status was included as a variable, which showed that case-family members were 6.36 times as likely as control family members to develop any SUD diagnosis over time (Hazard ratio (HR) = 6.36, p<0.001, 95% CI = 5.50–7.36). The results in the total sample also indicated that year of birth and males were significant. The hazard ratio (HR) showed, that being male (HR = 1.70, p<0.001, 95% CI = 1.49–1.94) was a risk factor, but that living outside of Copenhagen lowered the risk (HR = 0.74, p<0.001, 95% CI = 0.63–0.87). Later year of birth i.e. younger age (HR = 0.98, p<0.001, 95% CI = 0.97–0.99) also lowered the risk, but only very little. Furthermore, birth in August (HR = 1.42, p<0.05, 95% CI = 1.02–1.99) or September (HR = 1.51, p<0.05, 95% CI = 1.08–2.10) also had significant p-values, but the 95% confidence intervals for these two months were close to 1 and very similar to the confidence intervals for the other non-significant months, which indicated that it was no riskier to be born in August or September compared to January. The variance of the frailty was 1.96, which was not significant, i.e. the risk level of SUD in each family was almost explained by the included variables, which leaves no significant effect from unknown variables.

**Table 7:** Cox regression with shared frailty considering the time to diagnosis of SUD.

	TOTAL			ALC			MULT		
	HR	p	95% CI	HR	p	95% CI	HR	p	95% CI
Case/Control status									
Control	1.00		Reference	1.00		Reference	1.00		Reference
Case	6.36	<0.001	5.50–7.36	4.58	<0.001	3.34–6.30	10.32	<0.001	7.75–13.74
Sex									
Female	1.00		Reference	1.00		Reference	1.00		Reference
Male	1.70	<0.001	1.49–1.94	1.56	<0.05	1.15–2.12	1.63	<0.001	1.27–2.09
Month of birth									
January	1.00		Reference	1.00		Reference	1.00		Reference
February	1.07	n.s.	0.75–1.53	1.06	n.s.	0.47–2.38	0.91	n.s.	0.48–1.72
March	1.26	n.s.	0.90–1.76	1.11	n.s.	0.51–2.45	0.78	n.s.	0.42–1.46
April	1.14	n.s.	0.81–1.61	1.33	n.s.	0.62–2.88	0.75	n.s.	0.40–1.40
May	1.21	n.s.	0.86–1.70	1.81	n.s.	0.86–3.81	0.91	n.s.	0.50–1.67
June	1.14	n.s.	0.80–1.61	1.19	n.s.	0.52–2.71	0.84	n.s.	0.44–1.62
July	1.02	n.s.	0.71–1.45	0.86	n.s.	0.36–2.06	0.89	n.s.	0.48–1.66
August	1.42	<0.05	1.02–1.99	1.52	n.s.	0.70–3.28	1.55	n.s.	0.87–2.75
September	1.51	<0.05	1.08–2.10	1.43	n.s.	0.66–3.09	1.49	n.s.	0.84–2.63
October	1.21	n.s.	0.86–1.71	1.45	n.s.	0.67–3.17	1.00	n.s.	0.54–1.86
November	1.15	n.s.	0.81–1.64	0.74	n.s.	0.29–1.86	1.27	n.s.	0.69–2.33
December	1.32	n.s.	0.94–1.85	1.07	n.s.	0.47–2.43	1.27	n.s.	0.69–2.31
Year of birth	0.98	<0.001	0.97–0.99	1.01	n.s.	0.99–1.02	1.03	<0.001	1.02–1.05
Region of residence									
City of Copenhagen	1.00		Reference	1.00		Reference	1.00		Reference
Other regions	0.74	<0.001	0.63–0.87	0.80	n.s.	0.57–1.12	0.51	<0.001	0.38–0.69
Variance of frailty	1.96	n.s.		1.18	n.s.		2.63	n.s.	

HR = Hazard ratio

In the ALC subgroup, the Cox model with shared frailty showed that case family members were 4.58 times as likely as control family members to develop ALC over time (Hazard ratio (HR) = 4.58, p<0.001, 95% CI = 3.34–6.30). The model also showed that being male (HR = 1.56, p<0.05, 95% CI = 1.15–2.12) was a risk factor. The variance of the random effect was 1.18, which was not significant. The Cox model for the MULT subgroup showed that case family members were 10.32 times as likely as control family members to develop MULT over time (Hazard ratio (HR) = 10.32, p<0.001, 95% CI = 7.75–13.74). Furthermore, later year of birth (HR = 1.03, p<0.001, 95% CI = 1.02–1.05) and being male (HR = 1.63, p<0.001, 95% CI = 1.27–2.09) were risk factors, but living outside of Copenhagen (HR = 0.51, p<0.001, 95% CI = 0.38–0.69) lowered the risk. The variance of the random effect was 2.63 and not significant.

## Discussion

The first major finding of the present study was that SUD occurred more often in relatives of case- probands than in relatives of controls; statistically higher proportions of affliction were observed in fathers, mothers, and siblings. In particular, case-family members were more than six times as likely as control family members to develop any SUD diagnosis over time. This finding of a high family aggregation of SUD is very much in line with prior evidence of family transmission reported in both clinical samples [1–3, 7] and large registry studies [4–6].

The findings also corroborate the relevance of comorbid disorders on various levels. First, the large majority of case-probands had comorbid disorders with mostly PERS followed by DEP, CD, and ANX, and less frequently by ED and BP. These findings are in accordance with previous studies based on clinical samples pointing to the co-occurrence of SUD with ANX or DEP [8, 11] eating disorders [15, 16] and externalizing disorders including CD [12, 17] but also with our previous studies using the same study design on ED [26] and phobic disorders [27].

In addition, the study also underlined the major role that comorbid disorders in other family members play in the transmission process. Having family members affected by DEP, ANX, PERS, or CD increased the risk of SUD in the case-probands. These findings based on a large national dataset corroborate those from other family studies indicating high rates of, for example, comorbid anxiety disorders among parents with SUD [13]; but also point to a more widespread relevance of further major mental disorders in the families of case-probands with SUD. In the family transmission process, both SUD per se and comorbid disorders in the family members played an eminent role. The contribution of family SUD was highly relevant irrespective of the case-proband showing pure or comorbid SUD. In both associations the RRR was high and of similar magnitude.

The analyses as to the transmission of comorbid disorders shows that if comorbid disorders were present in a family, then the case-proband was more likely to have comorbid SUD rather than pure SUD. The analyses also showed that the presence of both SUD and one or more of the diagnoses BP, DEP, ANX, ED, PERS, or CD in a family member actually lowered the risk of SUD in the case-probands, compared to what one would expect when looking at the separate risks of SUD in the family and one or more comorbid disorders in the family. This is because the interaction term was less than one in both the group of case-probands with pure SUD and in the group of case-probands with comorbid SUD. These different patterns of family aggregation have not been identified before as previous family studies have only studied comorbid disorders in isolation.

In addition to the family effects, the present study showed the effect of both further risk and protective factors. Both advanced maternal or paternal age (≥35 years) had no significant effect in the present study. The recognition of these markers of both a biological and an educational risk as significant risk factors for the development of offspring disorders has been inconsistent in our family studies that have used the same overall study design in terms of an advanced paternal age in case-probands with schizophrenia [22] and an advanced maternal age in case-probands with obsessive-compulsive disorders [25]. Whereas a recent Swedish registry study found that an even higher paternal age (45+) was associated with increased risk of psychiatric and academic morbidity, including substance use problems in the offspring [21]. In contrast, male sex was a clear risk factor supporting the findings from most epidemiological surveys [18, 19]. Furthermore, the year of birth effect was too small to derive any solid conclusion.

Furthermore, there was a difference between the family load of case-probands with the diagnosis of SUD before age 18 vs. case-probands with the diagnosis of SUD at age 18 or later but before the end of the study. Both in the total group and in the ALC, but not in the MULT subgroup, the family load was higher in case-probands with a higher age at diagnosis of SUD. Thus, these findings lend no support to the vulnerability hypothesis in neurodevelopmental disorders that suggests that higher family loads of mental disorders contribute to an earlier manifestation of the respective disorder in the offspring. Such a higher family liability has, for instance, been put forward for schizophrenia but has not been observed in our own respective study [22].

Finally, the finding of a significant urbanization effect with an increased risk of living in the capital for the manifestation of SUD stands in line with findings from two our two previous studies using the same study design in case-probands affected by schizophrenia [22] or by ANX [24]. However, it is in contrast to no significant effects in case-probands with either BD [23], or obsessive-compulsive disorders [25], or ED [26], or phobic disorders [27]. Thus, there is no consistent pattern of findings on urbanization effects in this series of family studies. Similar mixed findings contributing to a meta-analysis of urban-rural differences in the prevalence of psychiatric disorders in population studies may have led to the overall finding of no urbanization effects for SUD [35].

However, after considering the various additional explanatory variables, the analyses indicated that the family load estimate explained 30 percent of the variance of the family aggregation of SUD, therefore implicating other important factors like co-morbid disorders in the etiology of the disease. This finding has to be considered in perspective with our other family aggregation studies that revealed a 23% rate of explained variance in schizophrenia [22], a 20% rate in bipolar disorders [23], a 12% rate in anxiety disorders [24], a 6% rate in obsessive compulsive disorders [25], but an almost zero effect in ED [26] and phobic disorders [27]. Thus, there is clear evidence that family load is higher in SUD in comparison to a large number of major psychiatric disorders. This reflects the strong effects of both genetic and environmental risk factors. Among the latter, socioeconomic status and neighborhood deprivation in particular, have been identified as risk factors of drug abuse in a large Swedish registry study [4].

Besides analyzing family aggregation and risk factors for developing SUD in the unselected total group, the two major subgroups of ALC and MULT were considered separately. By and large, the findings were identical or similar in these two subgroups. These findings highlight general risk factors of SUD rather than any drug-specific associations. However, it should be noted that the relatively large group of pure cannabinoid abusers among the case-probands was not suitable for analysis due to a relatively low family aggregation that may have been due to the fact that cannabinoid use had not yet been sufficiently prevalent in the parental generation. Furthermore, the rather small group of pure opioid abusers did not allow for separate analyses of these pure effects. However, both cannabinoid and opioid effects contributed to the effects observed in the MULT subgroup.

The finding of mostly lacking evidence of drug-specific associations stands in contrast to some previous studies based on smaller and less representative samples. For instance, the Collaborative Study on the Genetics of Alcoholism revealed evidence of both common and specific addictive factors transmitted in families for alcohol, marijuana, and cocaine dependence and habitual smoking and suggested independent causative factors in the development of each type of substance dependence [3]. Likewise, in a much smaller clinical cohort there was also evidence of specificity of familial aggregation of the predominant drug of abuse [7].

Advantages of the study include surveillance of a large population sample, an extended period of observation, a data set covering three generations and the matching of cases and controls on potentially confounding variables. There are several limitations. First, cases of illness for which treatment was not sought, or treated privately (although private care is uncommon in Denmark) were not included in the analysis. Secondly, there is no independent verification of the accuracy of diagnoses entered in the DCPRR, although prior quality checks on the DCPRR suggest that diagnostic validity is high across a range of disorders [36–39]. Thirdly, given that mental disorders in the data set are determined by treatment seeking, it can be assumed that many cases of familial illness remain “undetected,” and this may pertain to SUD as well. On the other hand, having a mentally ill relative might have increased the chance of seeking professional assistance and thus, the chance of registration rather than the risk of developing SUD. Fourthly, the rather late registration of outpatients in the DCPRR since 1995 may have resulted in an under-representation of the true number of patients with SUD. Finally, in the analyses the region of residence could only be defined as the hospital in which the case-probands received their first diagnosis.
